# A Tri-Component Conservation Strategy Reveals Highly Confident MicroRNA-mRNA Interactions and Evolution of MicroRNA Regulatory Networks

**DOI:** 10.1371/journal.pone.0103142

**Published:** 2014-07-23

**Authors:** Chen-Ching Lin, Ramkrishna Mitra, Zhongming Zhao

**Affiliations:** 1 Department of Biomedical Informatics, Vanderbilt University School of Medicine, Nashville, Tennessee, United States of America; 2 Department of Psychiatry, Vanderbilt University School of Medicine, Nashville, Tennessee, United States of America; 3 Department of Cancer Biology, Vanderbilt University School of Medicine, Nashville, Tennessee, United States of America; 4 Center for Quantitative Sciences, Vanderbilt University School of Medicine, Nashville, Tennessee, United States of America; Beijing Institute of Genomics, Chinese Academy of Sciences, China

## Abstract

MicroRNAs are small non-coding RNAs that can regulate expressions of their target genes at the post-transcriptional level. In this study, we propose a tri-component strategy that combines the conservation of microRNAs, homology of mRNA coding regions, and conserved microRNA binding sites in the 3′ untranslated regions to discover conserved microRNA-mRNA interactions. To validate the performance of our conservation strategy, we collected the experimentally validated microRNA-mRNA interactions from three databases as the golden standard. We found that the proposed strategy can improve the performance of existing target prediction algorithms by approximately 2–4 fold. In addition, we demonstrated that the proposed strategy could efficiently retain highly confident interactions from the intersection results of the existing algorithms and filter out the possible false positive predictions in the union one. Furthermore, this strategy can facilitate our ability to trace the homologues in different species that are targeted by the same miRNA family because it combines these three features to identify the conserved miRNA-mRNA interactions during evolution. Through an extensive application of the proposed conservation strategy to a study of the miR-1/206 regulatory network, we demonstrate that the target mRNA recruiting process could be associated with expansion of miRNA family during its evolution. We also uncovered the functional evolution of the miR-1/206 regulatory network. In this network, the early targeted genes tend to participate in more general and development-related functions. In summary, the conservation strategy is capable of helping to highlight the highly confident miRNA-mRNA interactions and can be further applied to reveal the evolutionary features of miRNA regulatory network and functions.

## Introduction

MicroRNAs (miRNAs) are small, highly conserved non-coding RNA molecules that are ∼22 nucleotides in length and are involved in numerous biological processes, such as development, differentiation, and growth [1]–[4]. By complementarily binding to target mRNA transcripts, miRNAs can trigger gene down-regulation or translational repression [5], [6]. So far, multiple algorithms have been developed for miRNA target prediction, and these algorithms vary from each other in their uses of additional refining strategies [4], [7]–[9]. For example, miRanda measures the thermodynamic stability between miRNAs and their putative target mRNAs [7], [8], [10], TargetScan searches the conserved seed pairing regions in the 3′ untranslated regions (UTRs) of genes using whole genome alignment [4], [11], and mimiRNA incorporates the expression profiles of miRNAs and mRNAs [9]. Among these algorithms, TargetScan has been reported to possess more robust prediction performance in various cellular systems [12]. One major reason for TargetScan’s superior execution is its utilization of conservation information across species, which can efficiently reduce the number of false positive predictions [13].

**Figure 1:**
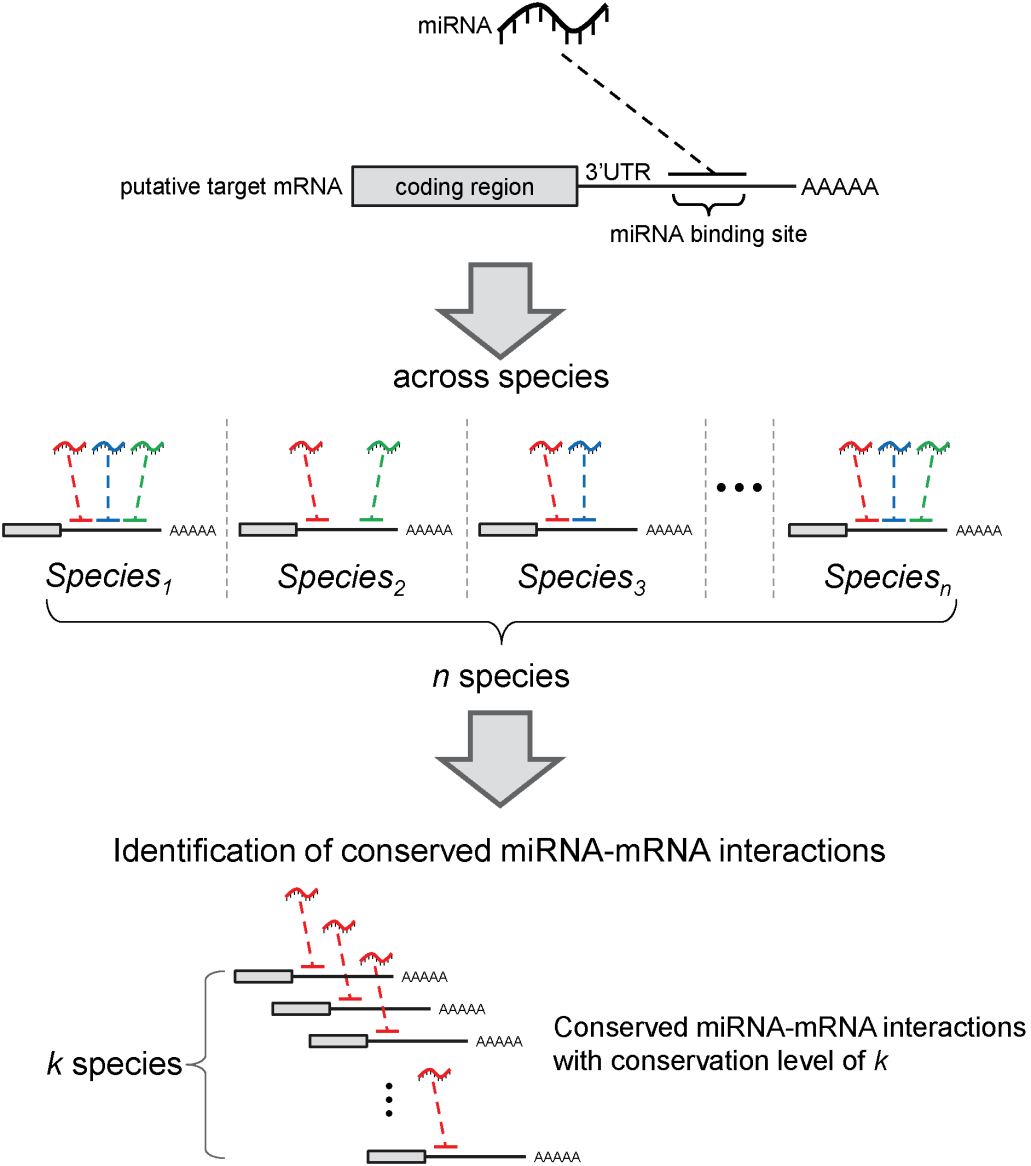
The tri-component conservation strategy scheme. The scheme of the proposed conservation strategy to identify the conserved miRNA-mRNA interactions is shown. The upper section shows the three major components of miRNA: the regulation-miRNA, mRNA coding region, and 3′ UTR of target mRNA. In the middle section, each color represents a member of one miRNA family. The putative target mRNAs are from homologues in each species. The lower section shows a miRNA-mRNA interaction conserved across *k* species. We further restricted the conserved miRNA-mRNA interactions that must be detected in both the oldest and youngest species; thus, *k* is from 2 to *n*.

Recently, the evolution of miRNAs has been studied extensively [14]–[18]. A miRNA is rarely lost during evolution once it has been established in a species [14]–[18]. The low secondary loss rate of miRNAs during evolution has been successfully applied to investigate the phylogeny of eukaryotic organisms [16], [18], [19]. Collectively, these studies indicated that the majority of miRNAs are highly conserved. Therefore, the conservation of miRNAs should be included in the identification of conserved miRNA-mRNA interactions. After reviewing several target prediction strategies, it became apparent that sequence conservation criteria in miRNA binding regions could increase overall precision and achieve better performance [12]. TargetScan reportedly possesses superior target prediction performance because of its utilization of conservation information; however, a high false positive miRNA-target prediction rate was also observed [20], [21]. Hence, an advanced conservation-based strategy that can accomplish improved target prediction performance is necessary. During miRNA evolution, the conserved miRNA-mRNA interactions may derive from the conservation traits of (1) miRNA, (2) coding region of target mRNA, and (3) miRNA binding sites in the 3′ UTR of the target mRNA. Therefore, an appropriate strategy to identify highly conserved miRNA-mRNA interactions should incorporate all three components into its algorithm to fully take into account the miRNA regulatory mechanisms. In this study, we proposed a conservation strategy to incorporate these three components into existing algorithms. This strategy combined miRNA conservation, mRNA coding region homology, and conserved miRNA binding sites in the 3′ UTRs into miRNA target predictions (Fig. 1). This conservation-based strategy was then used to discover the conserved miRNA-mRNA interactions at a large scale and investigate the evolution of the miRNA regulatory network and functions. Using the experimentally validated miRNA-mRNA interactions as the gold standard, we found that our strategy could improve the performance of the existing miRNA target prediction algorithms, including TargetScan. Finally, through an extensive application of our strategy to study the evolution of the miR-1/206 family, we demonstrated the evolutionary connections between this miRNA family and its regulatory network. Intriguingly, an evolutionary development (evo-devo) characteristic was observed in this network.

## Methods

### The sequences of mature miRNAs and 3′ UTR of mRNAs

The mature miRNA sequences from eight species, Caenorhabditis elegans, Drosophila melanogaster, Danio rerio, Xenopus tropicalis, Ornithorhynchus anatinus, Bos taurus, Mus musculus, and Homo sapiens, were obtained from miRBase Release 19 [22]. The 3′ UTR sequences of mRNAs in the above eight species and the homologous genes across species were obtained from Ensembl BioMart [23].

### MicroRNA-mRNA interactions

In this study, three algorithms, TargetScan [24]–[26], miRanda [8], and MultiMiTar [27], were used to predict the possible miRNA-mRNA interactions in eight species independently. These three algorithms use different information on miRNA target prediction. TargetScan focuses on seed complementary [22]–[24]; miRanda considers the thermodynamic properties between miRNA mature sequence and binding sites on target mRNA 3′ UTR [8]; MultiMiTar is a machine-learning based method that utilizes important miRNA-targeting specificity features from both the seed and out of seed interacting regions [27]. Besides using these three algorithms separately, we also built other three combinations of putative target gene pools from the above three algorithms. The first combination is the intersection of predicted miRNA-mRNA interactions from these three algorithms. However, the intersection would be biased by the minimum putative target set. Thus, the union, which collected all predicted results of these three algorithms, was considered to be the second combination data set. The intersection and union are believed to have reduced false positive and false negative prediction results respectively. To better utilize these two data sets, we created a combined miRNA-target mRNA set from the intersection and union as the third combination. This combination is termed as “IntSec(hsa),” which combines the intersection interactions in humans with the union of those interactions in the other seven species. In other words, IntSec(hsa) possessed the most strict predicted results in the species of interest, i.e. human, and the largest interaction set as evolutionary references in other species. In addition, IntSec(hsa) can be used to test if the proposed strategy is capable of filtering out the false positive reference interactions in other species while retaining the highly confident miRNA-mRNA interactions in the studied species.

### Experimentally validated miRNA-mRNA interactions

To assess the performance of the proposed conservation strategy, we compiled an experimentally validated miRNA-mRNA interaction dataset from the union of three databases, TarBase V5.0 [28], miRecords [29], and miRTarBase V4.4 [30], as the gold standard. Finally, 21,849 experimentally validated miRNA-mRNA interactions in humans were collected and used.

### The tri-component conservation strategy

In this study, we proposed a tri-component conservation strategy to discover conserved miRNA-mRNA interactions, to improve the performance of existing miRNA target prediction algorithms, and to investigate the evolution of miRNA regulatory networks (Fig. 1). This strategy combined the conservations of miRNAs, coding regions, and miRNA binding sites in the 3′ UTR. First, the evolutionarily conserved miRNA families were obtained from miRBase [22], [31]. For one miRNA family conserved across *n* species, there would be at least *n* member miRNAs. This step groups evolutionarily conserved miRNAs into families. In one species, one target mRNA set can be predicted for and assigned to a mature miRNA by an existing algorithm. Therefore, for one miRNA family conserved across *n* species, up to *n* number of target sets in *n* species can be predicted by one algorithm. Then, one target mRNA of the miRNA family *i* and its orthologues, which were predicted as target mRNAs of the miRNA family *i* in other species, were considered to be conserved target mRNAs of the miRNA family *i*. This step selects target mRNAs with conserved coding regions. Accordingly, our strategy required the conserved miRNA binding sites located in the homologue genes’ 3′ UTR and targeted by the members of one miRNA family. Consequently, the conserved miRNA-mRNA interactions with conserved miRNAs, target mRNA coding regions, and miRNA binding sites in the 3′ UTR can be identified by the tri-component conservation strategy.

Additionally, we further defined the conservation level of one conserved miRNA-mRNA interaction by the number of species in which this miRNA-mRNA interaction could be detected. For example, for a miRNA family with *n* species, a target mRNA that meets the criteria of the strategy in *k* species would be assigned a conservation level of *k*. To have further restriction, we required the conserved miRNA-mRNA interactions to be detected in both the oldest and youngest species; thus, *k* is from 2 to *n*.

## Results

### Improving miRNA target prediction using the tri-component conservation strategy

In this study, we developed a tri-component conservation strategy that combined the conservations of miRNAs, mRNA coding region, and miRNA binding sites in the 3′ UTR to predict highly conserved and confident miRNA-mRNA interactions (Fig. 1). This strategy was applied to three target prediction algorithms (TargetScan [24]–[26], miRanda [8], and MultiMiTar [27]) and three combination datasets (intersection, union, and IntSec(hsa)) across eight species (see Methods). Notably, the third combination dataset, IntSec(hsa), combines the intersection of three algorithms in humans and the union of three algorithms in the other seven species. Furthermore, a total of 21,849 experimentally validated miRNA-mRNA interactions in humans collected from three databases (TarBase V5.0 [28], miRecords [29], and miRTarBase V4.4 [30]) were used as the gold standard to evaluate the target prediction performance. The work-flow of our strategy was described in Figure S1.

**Figure 2:**
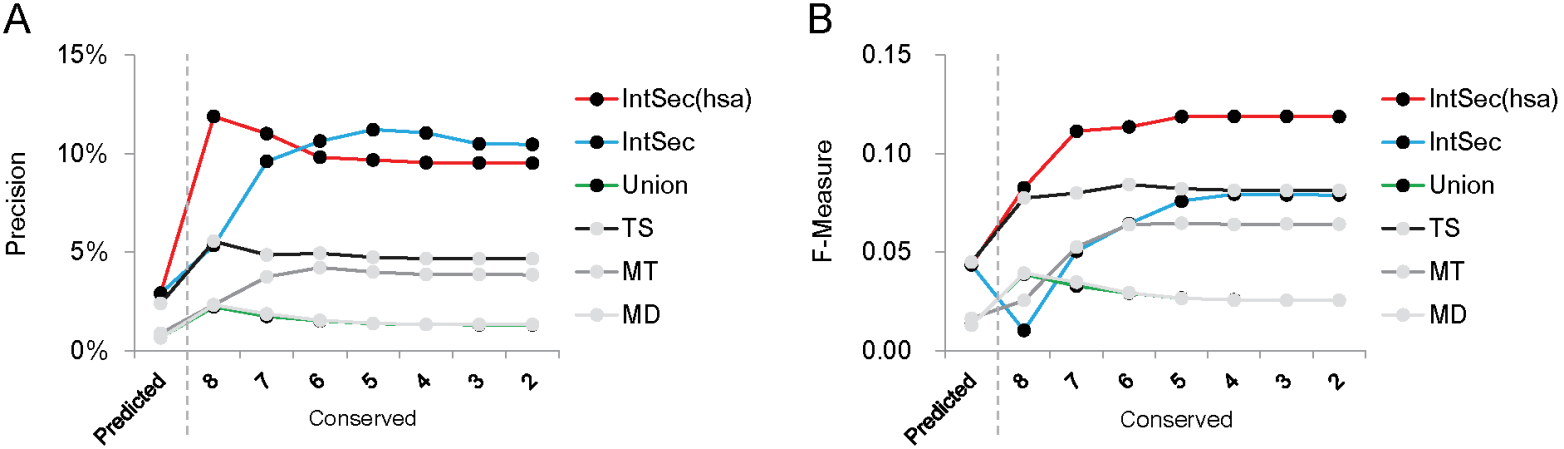
The improved performance of the conservation strategy. The performances of the conservation strategy and miRNA target prediction algorithms were evaluated by (A) precision and (B) F-measure. There are three algorithms (TS: TargetScan, MD: miRanda, and MT: MultiMiTar) and three combinations (IntSec: intersection, Union: union, and IntSec(hsa): intersection in humans with unions in other reference species). The results from the original algorithms/combinations were labeled “Predicted” (the left side of the dashed line). The results of the conserved miRNA-mRNA interactions identified by our strategy were labeled “Conserved” (the right side of the dashed line). The numbers along the X-axis indicate the conservation level of the conserved miRNA-mRNA interactions. Both the precision and F-measure are improved after applying the proposed conservation strategy. In two plots (2A and 2B), MD and union nearly overlap.

After applying our conservation strategy, the precision and F-measure values substantially increased by 2–4 fold compared to the original algorithms and combination data sets, i.e. intersection, union, and IntSec(hsa) (Fig. 2). F-measure, which is the harmonic mean of precision and recall, was used to assess the overall prediction performance in this study. This improvement indicated that our conservation strategy could efficiently identify highly confident (experimentally validated) miRNA-mRNA interactions from the original algorithms. Importantly, the conservation strategy in our study could improve the performance of TargetScan, which also incorporated conservation information into its own algorithm. However, TargetScan used the UTRs of the reference species based on orthology; that is, it used the aligned genomic regions between the reference species genome and the studied species based on whole genome alignment [24], [32]. In other words, the UTRs used by TargetScan in the reference species might not be a 3′ UTR of a gene. Different from TargetScan, our conservation strategy simultaneously considered the miRNA conservation, coding region homology, and conserved binding sites in the 3′ UTR of the (homologues) target mRNA. Accordingly, the overall improved performance elucidates that the underlying conservation strategy is useful to gain more confident results.

The conservation level of miRNA-mRNA interactions also affects the precision (Fig. 2). The conservation level is defined by the number of species in which this conserved miRNA-mRNA interaction can be detected. In most of the used data sets, as the conservation level decreased, the precision decreased and then became convergent after the conservation level of 6 (Fig. 2A). However, overall precision remained stronger than that of applying original algorithms only. This observation shows that the conservation strategy is very stable on predicting highly confident miRNA-mRNA interactions. In addition, this result also suggests that a higher conservation level could lead to a more precise prediction of the true miRNA-mRNA interactions. Notably, the F-measure of the intersection (IntSec) was dramatically decreased in the highest conservation level (Fig. 2B). This observation could be caused by overly stringent limitations on the intersection. However, the best F-measure was reached by applying our conservation strategy to IntSec(hsa). This observation also demonstrates that the conservation strategy can efficiently retain highly confident miRNA-mRNA interactions of the intersection in the studied species and filter out possible false positive predictions of the unions in other reference species.

### The evolution of the miR-1/206 family regulatory network: an extensive application of the tri-component conservation strategy

In contrast to the other target prediction algorithms, our proposed conservation strategy combined three major components (1) conservation of miRNAs (2) orthologues of target genes, and (3) conserved miRNA binding sites in the 3′ UTR. We combined these three features to identify the conserved miRNA-mRNA interactions during evolution. This strategy facilitated our ability to trace the homologues in different species that are targeted by the same miRNA family. Due to this intrinsic advantage, the conservation strategy can be further applied to study the evolution of miRNA regulatory networks. In this study, we used the miR-1/206 family to demonstrate this application. The combination putative target gene dataset of IntSec(hsa) was used to perform this analysis.

**Figure 3:**
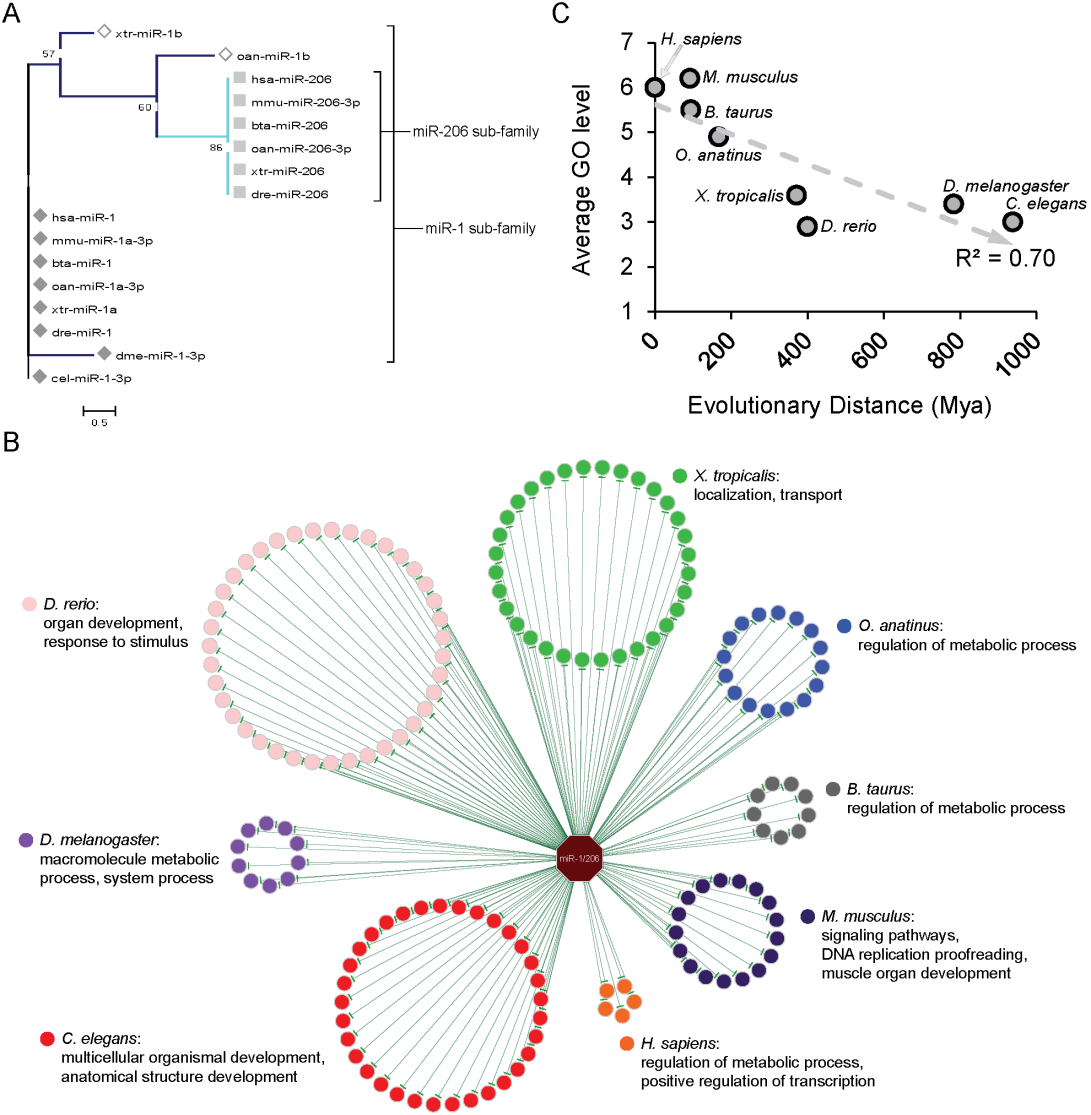
Evolutionary analyses of the miR-1/206 family regulatory network. (A) The phylogenetic tree of miR-1/206 family. This tree was drawn by MEGA 5.2.2 (Neighbor-Joining algorithm, 500 bootstrap replications) [42]. Blue: the branch of miR-1 subfamily; light blue: miR-206 subfamily. This tree shows that miR-1 subfamily existed before *C. elegans* and miR-206 subfamily before *D. rerio*. (B) The regulatory network of the miR-1/206 family in humans. The miR-1/206 family is represented by an octagon in the center of the network. Circles denote target genes of miR-1/206 in humans. Circle colors denote the most distant species in which the gene was targeted by the miR-1/206 family. The representative enriched functions specific to each species are listed under each species name. (C) The correlation between the Gene Ontology (GO) level of the top 10 enriched functions in miR-1/206 human target genes and the evolutionary distance. Target genes of older species tend to be enriched with more general biological functions, represented by lower levels of GO terms. (Mya: Million Years Ago).

MiR-1/206 is a highly conserved miRNA family from non-vertebrates to mammals (Fig. 3A) [18]. During its evolution, miR-1/206 branched into two subfamilies, miR-1 and miR-206 [33]. This observation of the highly similar mature sequences within each subfamily but relative dissimilarity between these two subfamilies (Fig. S2) warranted further investigation on their regulations, such as the gene networks regulated by these two subfamilies. Notably, the member miRNAs in the miR-1/206 family possess completely identical seed regions but different mature sequences (Fig. S2). Therefore, the miRNA-mRNA interactions predicted by the seed-based target prediction algorithms would be all the same between these two subfamilies. As a result, the evolution of miRNA-regulated networks between these two subfamilies can’t be observed. Accordingly, the combination target mRNA dataset is very proper to be used to discover the evolution of networks regulated by miRNAs in the same family. Therefore, we studied the miR-1/206 regulatory network in humans to discover the connections between the evolutions of the miR-1/206 family and its regulatory network. The human target genes identified by the conservation strategy were further grouped by the most distant (targeted) species in which their homologues were targeted by miR-1/206 (Fig. 3B). In general, the most distant species with homologues of a human gene is considered the species with the farthest evolutionary distance. In this study, with the intrinsic advantage of the conservation strategy, we extensively define the most distant (targeted) species of a human miRNA-target gene as the species with the most distant homologues targeted by the same miRNA family. Notably, the number of target gene was dramatically increased from *D. melanogaster* to *D. rerio* (increased by 2.9 fold, Fig. 3B and Fig. S3) when the miR-1/206 family branched to two subfamilies. This observation suggested that the variety of mature miRNA in one miRNA family could be reflected by the changes in its regulatory network during evolution. A previous study also reported that the size of miRNA family could affect the accumulation of their conserved target genes [34].

The functional evolution of the miR-1/206 regulatory network was investigated as well. Functions of genes were annotated with their biological process category in Gene Ontology (GO) [35]. For each gene group, the involved functions that have *P*≤0.05 as derived from the hypergeometric test were defined as significantly enriched. In addition, significantly enriched functions were ranked by the number of annotated genes, and the top 10 significantly enriched functions were listed in Table S1. The representative functions were summarized from the top 10 enriched biological processes in each gene group (Table S1) and labeled according to the corresponding gene group (Fig. 3B). We observed a series of variations in miR-1/206 regulatory functions during its evolution. The development-related functions first evolved in *C. elegans*, *D. melanogaster*, and *D. rerio*, and the functions involved in stimulus response also evolved in *D. rerio*. The cellular transport/localization-related functions then evolved in *X. tropicalis*. In *O. anatinus* and *B. taurus*, the miR-1/206 family evolved to regulate metabolic processes in cells. Additionally, signaling pathway-related biological processes and two more specific functions, DNA replication proofreading and muscle organ development, evolved in *M. musculus*. Finally, in *H. sapiens*, miR-1/206 regulatory functions evolved into positive regulations of transcription/gene expression. More importantly, we observed an association between the evolution of miR-1/206 regulatory network and its regulatory development-related functions. During the evolution of the miR-1/206 regulatory network, “multicellular organismal development” first evolved in *C. elegans.* This biological process participates in the developmental progression of a multicellular organism from its initial stage to late stage. “System process,” the function involved in the development of an organ system during a multicellular organismal process, evolved in *D. melanogaster*. Then, “organ development” and a more specific biological process, “muscle organ development,” evolved in *D. rerio* and *M. musculus*, respectively. Interestingly, the miR-1/206 family had been found to play a key role in the development of muscle organs [36]–[38]. These observations suggested that, from older to younger species, miR-1/206 regulatory developmental functions have evolved from a drastic to a mild level, i.e., from organismal level to organ-specific (i.e., muscle). In other words, an evo-devo feature of miR-1/206 regulatory functions was revealed by applying the proposed conservation strategy. Of note, the association between the evolution of miRNAs and the organismal complexity had been recently reported [39], [40]. Furthermore, investigating the GO level of enriched functions revealed that older target genes tend to be enriched in functions with a lower GO level (Fig. 3C). The evolutionary distances relative to *H. sapiens* were calculated by TimeTree [41] and represented with million years ago (Mya). In addition to using the top 10 enriched functions to perform the GO level analysis, analyses using the top 30, 20, and 10% enriched functions were also conducted and showed consistent conclusions (Fig. S4). In other words, early targeted genes of miR-1/206 family tend to participate in more general functions, and late ones tend to participate in more specific functions. However, genes with higher GO level might reflect more studies than those with lower GO level. To confirm this potential bias, we retrieved the number of publications for each gene from NCBI PubMed, which roughly reflects the extent of studies of the genes. We did not find the older targeted genes had more publications (Fig. S5). This preliminary analysis indicated no substantial bias on the extent of studies of each gene. In summary, this observation reconfirmed the evo-devo characteristic of miR-1/206 regulatory developmental functions from invertebrates to vertebrates and mammals.

## Discussion

In this study, we proposed a tri-component conservation strategy to identify the conserved miRNA-mRNA interactions and demonstrated its ability to improve the performance of existing target prediction algorithms. The improved performance of the proposed conservation strategy implies that conserved miRNA-mRNA interactions might be highly confident [12]. Even though the conservation strategy improved the performance of the three miRNA target prediction algorithms, its precision and F-measure are still relatively low. The highest precision is about 12% as reached by IntSec(hsa) at the most stringent conservation level of 8 (Fig. 2A), and the best F-measure is 0.12, also reached by IntSec(hsa), at a moderate conservation level of 5 (Fig. 2B). The low F-measure might indicate a relatively higher false negative rate in our strategy. The inadequate performance may result from the small and incomplete experimentally validated miRNA-mRNA interaction dataset. To confirm this, we removed those miRNAs with <200 experimentally validated targets and re-calculated the precision. We found that the highest precision achieved 37% by IntSec(hsa) with the most stringent conservation level of 8. Using a pooled miRNA data set (miR-1, miR-30, miR-155, miR-16, and let-7b), Selbach *et al*. [12] reported the precision of their miRNA target prediction approach, pSILAC, was approximately 30–60%. Interestingly, the precision of IntSec(hsa) was 56% when using the same miRNA data set. These observations further confirmed our explanations and pointed out that the proposed strategy might be capable of obtaining highly confident miRNA-mRNA interactions from the existing prediction algorithms. The best performance was observed for IntSec(hsa). IntSec(hsa) combines the intersection target gene set in humans and the union in other species. The intersection dataset was the smallest with expectation to possess a high precision rate, while the union created the largest dataset with a high recall rate. In other words, IntSec(hsa) integrated the smallest but highly confident target set in humans with the target sets as large as possible in other species as reference. This combination achieved the best performance on predicting experimentally validated miRNA-mRNA interactions. Thus, this observation indicated that the conservation strategy had a robust trade-off between precision and recall. Moreover, through our strategy, the IntSec(hsa) could take advantages of both the intersection and union. The results of MD and the union datasets were almost the same (Fig. 2A and 2B), suggesting that the union dataset was dominated by the prediction results of MD. Additionally, species-specific miRNA-mRNA interactions might be omitted by the innate manipulation of the conservation strategy. This shortcoming could be improved by using a group of closely related species as a reference (e.g., using mammals or primates as the references to predict miRNA-mRNA interactions in humans). Briefly, our conservation strategy improves the performance of predicting highly confident miRNA-mRNA interactions. In addition, we applied the conservation strategy to study the evolution of the miR-1/206 family. This extensive application further revealed the evolutionary connections between the miR-1/206 family and its regulatory network and demonstrated the functional evolution of the miR-1/206 regulatory network.

## Supporting Information

Figure S1 **The work-flow of the tri-component conservation strategy.** First, we obtained the mature miRNA sequences from miRBase 19 and 3′ UTR sequences from Ensembl BioMart for eight studied species. With the above two datasets, we run three existing target prediction algorithms [8], [24]–[27] to produce putative miRNA-mRNA interactions (MMIs) for one studied species. In this study, human is the studied species. Consequently, for each species, we obtained three putative MMI sets from three existing algorithms. Furthermore, two combinational MMI sets, i.e., intersection and union, have been obtained. Next, we executed this target prediction process on eight studied species. After this step, there would be eight putative MMI sets for each algorithm or each combinational dataset. Next, we created IntSec(hsa) that was consisted of the intersection MMIs in humans and the union ones in the other seven species. We denoted these six MMI sets, i.e. TargetScan, miRanda, MultiMiTar, intersection, union, and IntSec(hsa), as combinations. Until here, we obtained eight putative MMI sets for each combination. Furthermore, for eight species, we obtained miRNA family from miRBase [22] and homologues information from Ensembl BioMart [23], respectively. The member miRNAs in one miRNA family are evolutionary conserved. Then, for each combination, we grouped putative target genes into homologues target gene sets across eight species. The MMIs, formed by genes in homologues target gene set and the member miRNAs of one miRNA family in different species, have been identified as the conserved MMIs of the corresponding miRNA family. The strategy was depicted in Fig. 1. Furthermore, the number of species in which the conserved MMI was formed has been denoted as its conservation level of the observed conserved target genes. To have further restriction, we required the conserved MMIs to be detected in both the oldest and youngest species of the homologues target gene set. Finally, we compiled an experimentally validated MMI set from the union of three databases, TarBase V5.0 [28], miRecords [29], and miRTarBase V4.4 [30]. Using this MMI set as the gold standard, we can evaluate the performance of each MMI combination.
(TIF)

Figure S2 **The mature sequences of miR-1/206 family.** This figure shows the mature sequences of miR-1/206 family. The background colors represented the different types of nucleotides. The RNAs in seed regions were colored in white.
(TIF)

Figure S3 **The size variety of miR-1/206 regulatory network during evolution.** The human target gene sizes in the most distant species were shown at y-axis. There is a dramatic increasing of target gene size in between *D. melanogaster* and *D. rerio*.
(TIF)

Figure S4 **The correlation between the evolutionary distance and GO level.** The correlation that older target genes tend to be enriched in lower level GO functions was further confirmed by other three criteria, top 20, 30, and 10%. (Mya: Million Years Ago).
(TIF)

Figure S5 **The correlation between the evolutionary distance and the number of literatures.** The correlation that older target genes don’t tend to be studied more was further confirmed. (Mya: Million Years Ago).
(TIF)

Table S1 **The top 10 enriched functions in the most distant species.**
(PDF)
