# Supplementary figures and images for: A Tri-Component Conservation Strategy Reveals Highly Confident MicroRNA-mRNA Interactions and Evolution of MicroRNA Regulatory Networks
Source: PLoS One. 2014 Jul 23;9(7):e103142. doi: 10.1371/journal.pone.0103142 (PMC4108425; doi:10.1371/journal.pone.0103142)

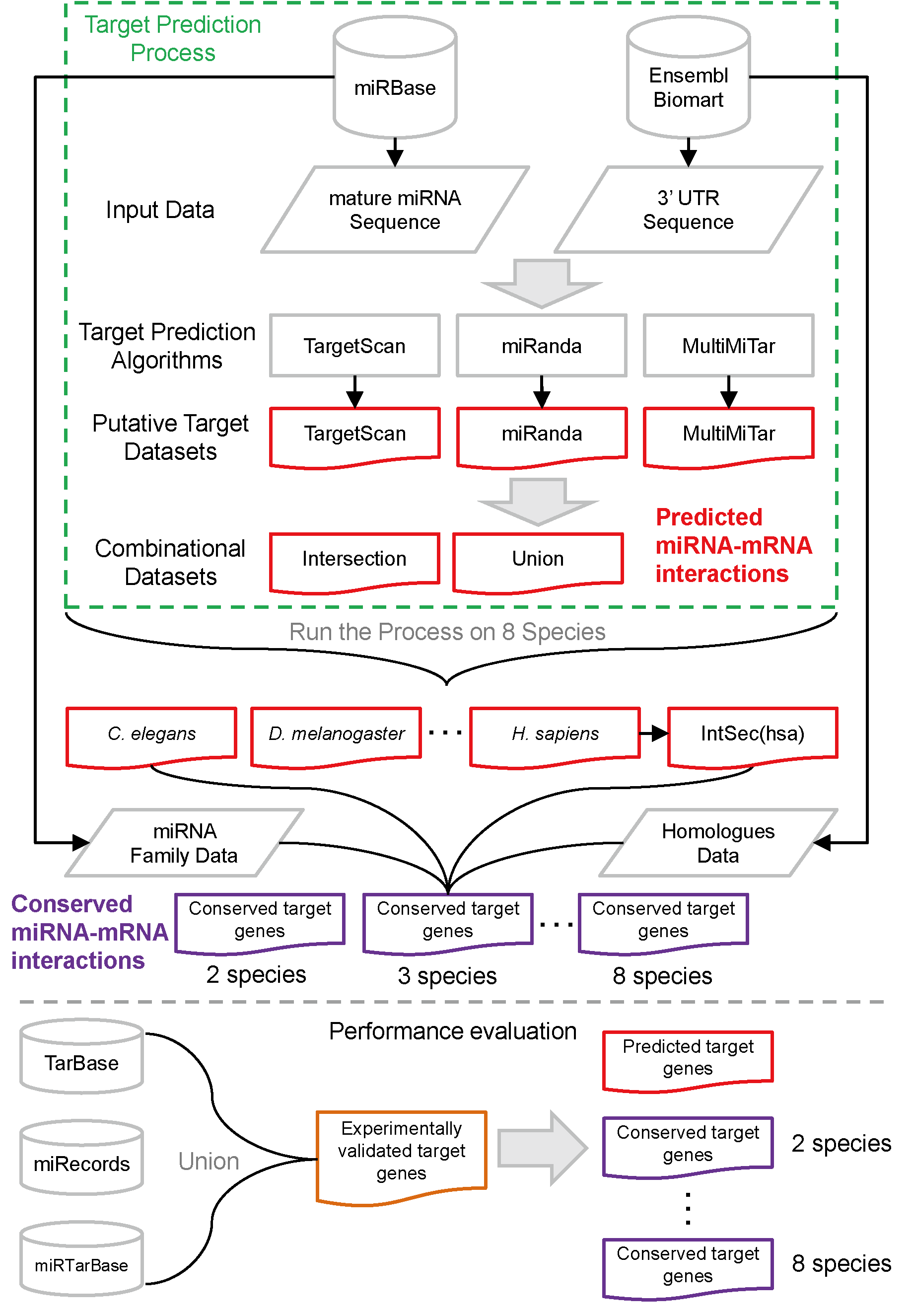

Supplement: Figure S1 — The work-flow of the tri-component conservation strategy. First, we obtained the mature miRNA sequences from miRBase 19 and 3′ UTR sequences from Ensembl BioMart for eight studied species. With the above two datasets, we run three existing target prediction algorithms [8], [24]–[27] to produce putative miRNA-mRNA interactions (MMIs) for one studied species. In this study, human is the studied species. Consequently, for each species, we obtained three putative MMI sets from three existing algorithms. Furthermore, two combinational MMI sets, i.e., intersection and union, have been obtained. Next, we executed this target prediction process on eight studied species. After this step, there would be eight putative MMI sets for each algorithm or each combinational dataset. Next, we created IntSec(hsa) that was consisted of the intersection MMIs in humans and the union ones in the other seven species. We denoted these six MMI sets, i.e. TargetScan, miRanda, MultiMiTar, intersection, union, and IntSec(hsa), as combinations. Until here, we obtained eight putative MMI sets for each combination. Furthermore, for eight species, we obtained miRNA family from miRBase [22] and homologues information from Ensembl BioMart [23], respectively. The member miRNAs in one miRNA family are evolutionary conserved. Then, for each combination, we grouped putative target genes into homologues target gene sets across eight species. The MMIs, formed by genes in homologues target gene set and the member miRNAs of one miRNA family in different species, have been identified as the conserved MMIs of the corresponding miRNA family. The strategy was depicted in Fig. 1. Furthermore, the number of species in which the conserved MMI was formed has been denoted as its conservation level of the observed conserved target genes. To have further restriction, we required the conserved MMIs to be detected in both the oldest and youngest species of the homologues target gene set. Finally, we compiled an experimen [file pone.0103142.s001.tif]

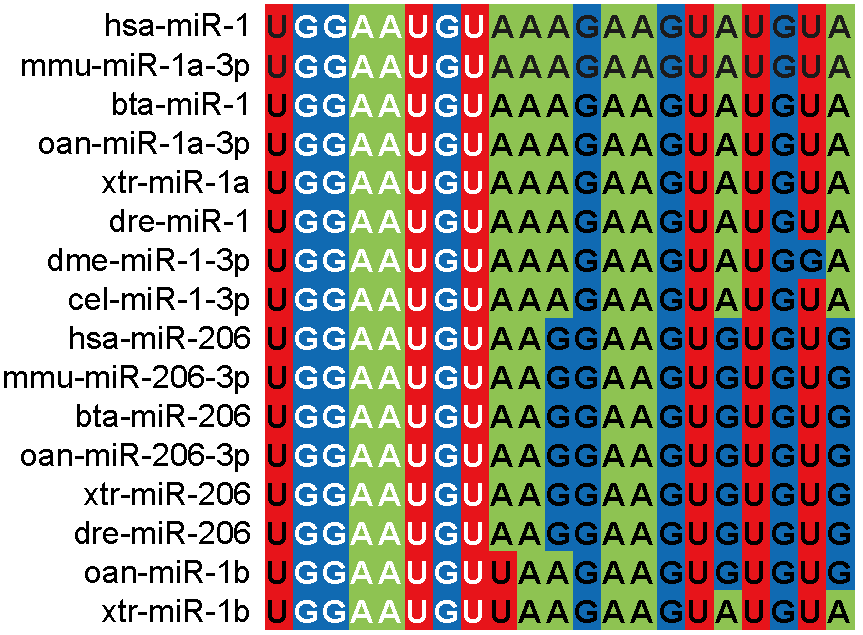

Supplement: Figure S2 — The mature sequences of miR-1/206 family. This figure shows the mature sequences of miR-1/206 family. The background colors represented the different types of nucleotides. The RNAs in seed regions were colored in white. (TIF) [file pone.0103142.s002.tif]

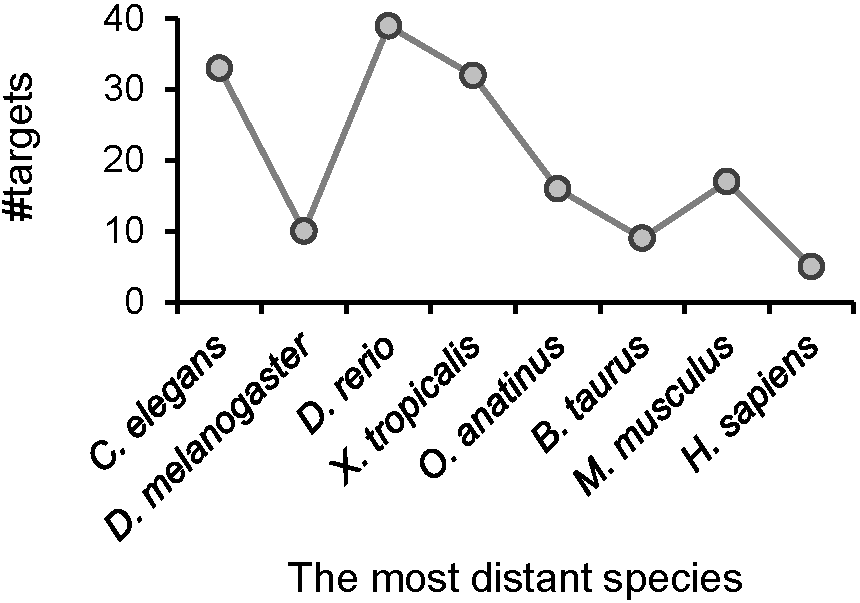

Supplement: Figure S3 — The size variety of miR-1/206 regulatory network during evolution. The human target gene sizes in the most distant species were shown at y-axis. There is a dramatic increasing of target gene size in between D. melanogaster and D. rerio. (TIF) [file pone.0103142.s003.tif]

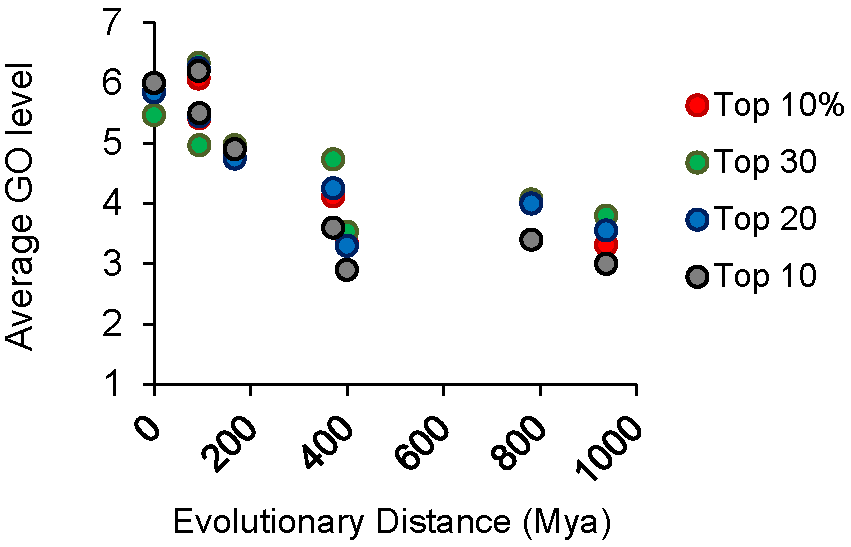

Supplement: Figure S4 — The correlation between the evolutionary distance and GO level. The correlation that older target genes tend to be enriched in lower level GO functions was further confirmed by other three criteria, top 20, 30, and 10%. (Mya: Million Years Ago). (TIF) [file pone.0103142.s004.tif]

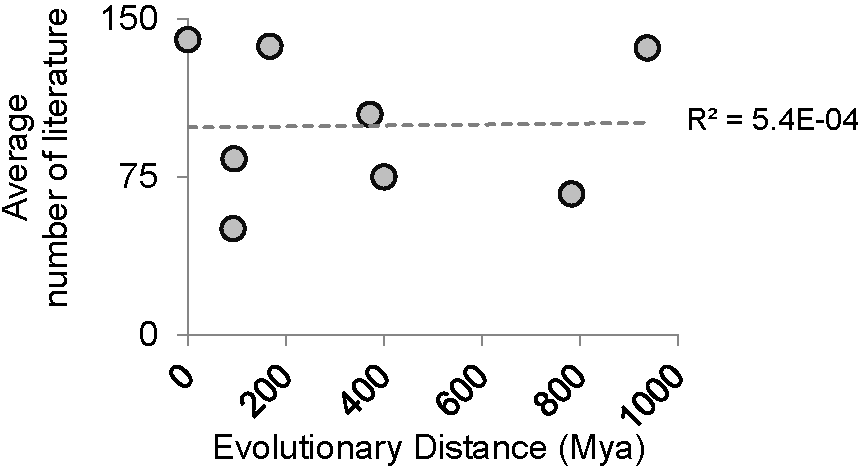

Supplement: Figure S5 — The correlation between the evolutionary distance and the number of literatures. The correlation that older target genes don’t tend to be studied more was further confirmed. (Mya: Million Years Ago). (TIF) [file pone.0103142.s005.tif]
