# Supplementary material for: A Tri-Component Conservation Strategy Reveals Highly Confident MicroRNA-mRNA Interactions and Evolution of MicroRNA Regulatory Networks
Source: PLoS One. 2014 Jul 23;9(7):e103142. doi: 10.1371/journal.pone.0103142 (PMC4108425; doi:10.1371/journal.pone.0103142)
Supplement: Table S1 — The top 10 enriched functions in the most distant species. (PDF) [file pone.0103142.s006.pdf]

Table S1: The top 10 enriched functions in the most distant species.

Level	GO Term	Description	Targets(%)	*p*-value
		C. elegans		
3	GO:0044707	single-multicellular organism process	58%	0.01
2	GO:0032501	multicellular organismal process	58%	0.01
2	GO:0032502	developmental process	48%	0.01
5	GO:0048522	positive regulation of cellular process	45%	< 0.01
3	GO:0044767	single-organism developmental process	45%	< 0.01
4	GO:0048518	positive regulation of biological process	45%	0.01
3	GO:0048856	anatomical structure development	45%	0.01
3	GO:0007275	multicellular organismal development	45%	0.01
3	GO:0016043	cellular component organization	45%	0.01
		D. melanogaster		
3	GO:0044707	single-multicellular organism process	80%	< 0.01
2	GO:0032501	multicellular organismal process	80%	0.01
4	GO:0043170	macromolecule metabolic process	80%	0.02
4	GO:0019538	protein metabolic process	60%	0.01
3	GO:0048856	anatomical structure development	60%	0.02
3	GO:0007275	multicellular organismal development	60%	0.02
2	GO:0032502	developmental process	60%	0.04
4	GO:0003008	system process	50%	< 0.01
5	GO:0048523	negative regulation of cellular process	50%	0.03
		D. rerio		
2	GO:0050896	response to stimulus	67%	0.01
2	GO:0032501	multicellular organismal process	58%	0.01
3	GO:0007275	multicellular organismal development	55%	< 0.01
2	GO:0032502	developmental process	55%	< 0.01
3	GO:0044707	single-multicellular organism process	55%	0.01
4	GO:0019222	regulation of metabolic process	52%	0.03
3	GO:0051716	cellular response to stimulus	52%	0.04
3	GO:0044767	single-organism developmental process	48%	< 0.01
3	GO:0048856	anatomical structure development	48%	< 0.01
		X. tropicalis		
2	GO:0051179	localization	45%	0.02
3	GO:0006810	transport	41%	0.01
2	GO:0051234	establishment of localization	41%	0.01
3	GO:0044765	single-organism transport	34%	0.02
4	GO:0071702	organic substance transport	31%	< 0.01
3	GO:0033036	macromolecule localization	28%	0.01
5	GO:0043436	oxoacid metabolic process	24%	< 0.01
4	GO:0006082	organic acid metabolic process	24%	< 0.01
6	GO:0032268	regulation of cellular protein metabolic process	24%	0.01

Table S1 (Cont.): The top 10 enriched functions in the most distant species.

Level	GO Term	Description	Targets(%)	*p*-value
		O. anatinus		
5	GO:0060255	regulation of macromolecule metabolic process	71%	< 0.01
5	GO:0080090	regulation of primary metabolic process	71%	< 0.01
5	GO:0031323	regulation of cellular metabolic process	71%	< 0.01
4	GO:0019222	regulation of metabolic process	71%	0.01
4	GO:0043170	macromolecule metabolic process	71%	0.03
6	GO:0010604	positive regulation of macromolecule metabolic process	57%	< 0.01
6	GO:0031325	positive regulation of cellular metabolic process	57%	< 0.01
5	GO:0009893	positive regulation of metabolic process	57%	< 0.01
5	GO:0048522	positive regulation of cellular process	57%	< 0.01
		B. taurus		
5	GO:0060255	regulation of macromolecule metabolic process	86%	< 0.01
5	GO:0080090	regulation of primary metabolic process	86%	< 0.01
5	GO:0031323	regulation of cellular metabolic process	86%	< 0.01
4	GO:0019222	regulation of metabolic process	86%	0.01
7	GO:0031399	regulation of protein modification process	71%	< 0.01
6	GO:0032268	regulation of cellular protein metabolic process	71%	< 0.01
6	GO:0051246	regulation of protein metabolic process	71%	< 0.01
6	GO:0010604	positive regulation of macromolecule metabolic process	71%	< 0.01
6	GO:0031325	positive regulation of cellular metabolic process	71%	< 0.01
		M.musculus		
6	GO:0007167	enzyme linked receptor protein signaling pathway	20%	0.03
4	GO:0005975	carbohydrate metabolic process	20%	0.04
7	GO:0038165	oncostatin-M-mediated signaling pathway	13%	< 0.01
7	GO:0048861	leukemia inhibitory factor signaling pathway	13%	< 0.01
7	GO:0070120	ciliary neurotrophic factor-mediated signaling pathway	13%	< 0.01
6	GO:0005996	monosaccharide metabolic process	13%	0.02
5	GO:0007517	muscle organ development	13%	0.03
7	GO:0038154	interleukin-11-mediated signaling pathway	7%	< 0.01
6	GO:0045004	DNA replication proofreading	7%	< 0.01
		H. sapiens		
6	GO:0019219	regulation of nucleobase-containing compound metabolic process	80%	0.01
5	GO:0051171	regulation of nitrogen compound metabolic process	80%	0.02
5	GO:0080090	regulation of primary metabolic process	80%	0.03
5	GO:0031323	regulation of cellular metabolic process	80%	0.04
6	GO:0045944	positive regulation of transcription from RNA polymerase II promoter	60%	< 0.01
6	GO:0045893	positive regulation of transcription, DNA-dependent	60%	< 0.01
7	GO:0051254	positive regulation of RNA metabolic process	60%	< 0.01
6	GO:0010628	positive regulation of gene expression	60%	< 0.01
7	GO:0045935	positive regulation of nucleobase-containing compound metabolic process	60%	< 0.01
